# Magnetic resonance imaging–based classification of cesarean scar pregnancy: prediction of intraoperative blood loss and the role of preoperative uterine artery embolization

**DOI:** 10.3389/fmed.2026.1734573

**Published:** 2026-02-09

**Authors:** Xiang Li, Tiankuan Li, Mujun Dong, Jiahuan Liu, Fei Wang, Xiaojun Yang

**Affiliations:** 1 Department of Radiology, Quzhou People’s Hospital, The Quzhou Affiliated Hospital of Wenzhou Medical University, Quzhou, Zhejiang, China; 2 Department of Radiology, Ruijin Hospital, Shanghai Jiao Tong University School of Medicine, Shanghai, China; 3 Department of Radiology, Quzhou Maternal and Child Health Care Hospital, Quzhou, Zhejiang, China

**Keywords:** cesarean scar pregnancy, dilation and curettage, intraoperative hemorrhage, magnetic resonance imaging, uterine artery embolization

## Abstract

**Objectives:**

To establish a magnetic resonance imaging (MRI)–based classification system for cesarean scar pregnancy (CSP), assess its ability to predict intraoperative blood loss, and evaluate the effectiveness of prophylactic uterine artery embolization (UAE).

**Methods:**

Ninety-eight women diagnosed with CSP who underwent MRI evaluation between May 2016 and September 2023, and CSP was classified into three subtypes according to sac morphology, vascular characteristics, and scar myometrial thickness. Subgroups were further stratified by preoperative UAE status. Intraoperative blood loss during pregnancy termination, evaluated by regression analysis and intergroup comparison.

**Results:**

Thirty-five patients were classified as type I, 35 as type II, and 28 as type III. Type I CSPs typically showed simple cystic sacs with minimal vascularity, while type II exhibited moderate vascularity and mixed cystic-solid features. Type III was characterized by large mixed cystic-solid sacs with prominent vascular flow voids, vascular lakes, and arteriovenous fistulas. Median intraoperative blood loss was 20 mL for type I, 50 mL for type II, and 265 mL for type III (*p* < 0.001). Multiple linear regression confirmed type III as the strongest independent predictor of hemorrhage (β = 327.2, *p* < 0.001). Among type III patients, preoperative UAE significantly reduced blood loss (*p* < 0.001), whereas, based on the limited data from this study, no significant benefit of preoperative UAE was observed in patients with type I or type II.

**Conclusion:**

Magnetic resonance imaging classification provides a reliable framework to stratify hemorrhage risk in CSP. Type III is associated with substantial intraoperative bleeding, and preoperative UAE is highly effective in mitigating this risk. Incorporating MRI classification into routine assessment may guide individualized management and improve surgical outcomes.

## 1 Introduction

Cesarean scar pregnancy (CSP), also referred to as cesarean scar ectopic pregnancy, is a rare type of ectopic pregnancy in which a fertilized ovum implants within the scar of a previous cesarean section (1). The global incidence of CSP is increasing, estimated at 1/1,800 to 1/2,500 pregnancies, and affecting up to 1.15% of women with a prior cesarean delivery (2). This trend is strongly associated with the rising rate of cesarean sections worldwide.

A prior cesarean section is the primary risk factor for CSP. Surgical disruption of the lower uterine segment may lead to a poorly healed scar with myometrial thinning or defect, creating a potential niche for abnormal embryo implantation (3). Other uterine surgeries—such as myomectomy or repeated curettage—also increase CSP risk by compromising the structural integrity of the myometrium (4). Multiple prior cesarean deliveries have a cumulative effect, significantly increasing the risk of CSP (5).

Delayed diagnosis and non-standardized treatment can lead to serious complications. The thin, highly vascularized myometrium at the scar site predisposes patients to massive hemorrhage during curettage, with reported rates ranging from 11% to 59.3% (6). As pregnancy advances, risks escalate to include uterine rupture, placenta accreta spectrum (PAS), and involvement of adjacent organs such as the bladder. In severe cases, hysterectomy may be necessary, resulting in permanent loss of fertility. Early, accurate diagnosis and tailored intervention are therefore essential to improve patient outcomes (7).

Uterine artery embolization (UAE) has emerged as a valuable minimally invasive intervention for CSP. By embolizing branches of the uterine artery, UAE reduces blood supply to the implantation site, significantly lowering intraoperative blood loss, shortening surgical duration, and reducing the risk of hysterectomy (8). For patients with high-risk CSP, a condition inherently linked to severe maternal complications such as massive hemorrhage and uterine rupture, preoperative uterine artery embolization (UAE) is a vital intervention. UAE has been demonstrated to reduce the incidence of severe hemorrhage by over 60%, effectively enhancing patient safety and promoting fertility preservation (9). UAE is increasingly adopted as a pretreatment strategy for CSP patients at substantial risk of bleeding.

Ultrasound (transabdominal or transvaginal) is the first-line modality due to its accessibility, real-time capabilities, and absence of radiation. It allows for detailed assessment of the gestational sac’s location, myometrial thickness, and surrounding vascularity. Diagnostic criteria include an empty uterine cavity and cervical canal, a gestational sac embedded in the lower anterior uterine wall at the scar site, thinning or disruption of the myometrium between the sac and the bladder, and high-velocity, low-resistance peri trophoblastic flow on Doppler imaging (10). While US is excellent for screening and measuring the residual myometrial thickness, MRI’s superior soft-tissue contrast allows for a more comprehensive, multi-planar, and non-invasive assessment of the gestational sac’s precise depth and three-dimensional extent of invasion into the myometrium and adjacent structures, particularly the posterior wall of the bladder. This detailed anatomical relationship is often indeterminate by US and is critical for planning complex individualized procedures. Moreover, ultrasound diagnosis can be limited by operator experience, pelvic adhesions, and bowel gas, with atypical cases (e.g., indistinct sac-scar boundaries or concurrent hematoma) carrying a missed diagnosis rate of 15%–20% (7).

Magnetic resonance imaging (MRI) offers superior soft tissue contrast and multiplanar imaging, complementing ultrasound in complex cases. MRI allows for precise evaluation of the relationship between the gestational sac, uterine scar, bladder, and myometrium, and can assess the depth of invasion and vascular involvement (6). In cases where ultrasound findings are inconclusive—such as mixed echo masses or suspected uterine rupture—MRI can achieve diagnostic accuracy exceeding 90% (10). Existing classification systems are primarily ultrasound-based, focusing on parameters such as myometrial thickness and sac size, with no standardized MRI framework to guide treatment selection.

In this study, we retrospectively analyzed clinical and imaging data from 98 CSP patients treated at our institution, and developed a novel MRI-based classification system. Based on MRI features—including scar myometrial thickness, gestational sac size, invasion extent, and presence of arteriovenous fistula. We investigated the association between classification type and intraoperative blood loss during curettage and evaluated the efficacy of preoperative UAE in reducing bleeding across classification groups. Our findings aim to support individualized treatment planning, improve management of high-risk cases, and reduce CSP-related complications.

## 2 Materials and methods

### 2.1 Diagnostic criteria for cesarean scar pregnancy

The diagnostic criteria for CSP are as follows: Amenorrhea, complicated with or without irregular vaginal bleeding; Elevated serum β-human chorionic gonadotropin (β-hCG) levels; A history of previous cesarean section or uterine surgery. Typical findings on magnetic resonance imaging (MRI) or ultrasonography: (1) The uterine cavity and cervical canal are empty, with no gestational sac detected; (2) The gestational sac is implanted in the myometrium of the lower anterior uterine segment (corresponding to the site of the previous cesarean section incision), and some gestational sacs may contain fetal buds or exhibit fetal cardiac activity; (3) The continuity of the anterior uterine myometrium is disrupted, and the myometrium between the gestational sac and the bladder is significantly thinned or even absent; (4) MRI or color Doppler flow imaging (CDFI) reveals blood flow signals around the gestational sac.

### 2.2 Study sample

**FIGURE 1:**
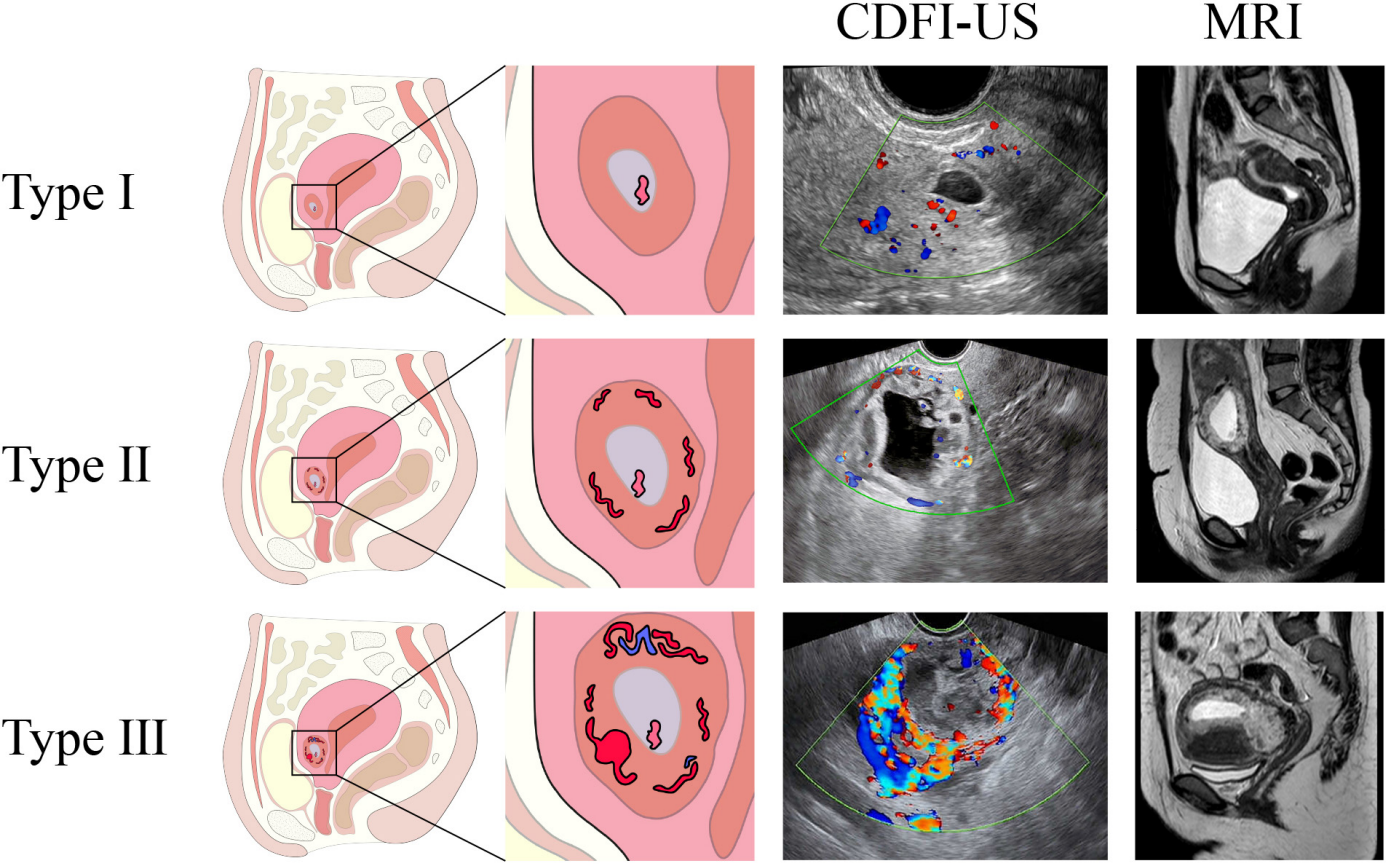
Schematic diagrams of the three types. Type I: The gestational sac is regular, with poor blood supply around it. Type II: There is relatively abundant blood supply around the gestational sac, with visible flow-void vascular shadows around it. Type III: There is rich blood supply around the gestational sac, with visible vascular lakes and arteriovenous fistulas.

This retrospective clinical study was approved by the Quzhou People’s Hospital Ethics Committee. A total of 98 patients with cesarean scar pregnancy (CSP) who underwent MRI examination at our hospital from May 2016 to September 2023 were included. Exclusion criteria were: history of contrast agent allergy, uterine rupture, hemorrhagic shock, severe coagulation disorders, and severe comorbidities involving major organs (heart, liver, brain, lungs, kidneys). According to MRI findings, patients were categorized into three types (Figure 1): Type I (Hypo vascular type): Gestational sac showed no obvious enhancement or non-enhancement. Type II: Gestational sac was enhanced, with a small amount of flow-void vascular shadows around it, but no obvious vascular lakes, arteriovenous fistulas, pseudoaneurysms, or aneurysms. Type III (High-risk type): Gestational sac showed significant enhancement, with surrounding flow-void vascular shadows, accompanied by one or more of the following: obvious vascular lakes, arteriovenous fistulas, pseudoaneurysms, or aneurysms. All patient MRI images were retrospectively classified independently by two senior radiologists, each possessing over 10 years of experience in abdominal and gynecological imaging diagnosis, thus ensuring a high level of expertise in the initial classification. In the initial classification phase, any cases where the two readers had a disagreement regarding the Type (e.g., borderline Type II/III cases concerning vascularity) were resolved through mutual discussion and consensus.

### 2.3 MRI examination method

Patients were required to fast for more than 4 h before the examination and drink an appropriate amount of water to achieve moderate bladder filling. If necessary, 10 mg of anisodamine was intramuscularly injected to reduce motion artifacts caused by intestinal peristalsis. Gadopentetate dimeglumine injection (0.1 mmol/kg) was administered via the cubital vein at a rate of 2 ml/s. After the injection, examinations were performed using a GE Signa Voyager 1.5T magnetic resonance scanner. First, sagittal scans were performed. Based on the sagittal images, axial and coronal scan planes were determined according to the relationship between the lesion and the scar. The main scanning sequences included: sagittal T2WI, sagittal T1WI, axial LAVA-Flex, coronal T2WI FSE with fat saturation, and axial DWI.

### 2.4 Uterine artery embolization

Verify the patient’s identity, confirm the puncture site, and establish intravenous access. The patient was placed in a supine position, and local anesthesia was administered. Puncture and catheterization were performed via the right/left femoral artery or left/right radial artery. Angiography with a pigtail catheter was performed at a level slightly above the renal artery plane of the abdominal aorta to observe the visualization of relevant responsible arteries and the lesion. Under fluoroscopic guidance, super-selective catheterization into the bleeding artery was performed, respectively and confirmed by angiography. Gelatin sponge particles were continuously injected until blood flow to the lesion ceased and the blood flow in the main trunk of the uterine artery slowed significantly. The puncture site was then dressed, and the operation was completed (Supplementary Figure 1).

### 2.5 Ultrasound-guided dilation & curettage (USg-D&C)

The patient is carefully placed in the lithotomy position. The surgical area is disinfected, sterile drapes are applied, and an ultrasound transducer is placed transabdominally to visualize the uterus. The physician gently inserts a curette into the uterine cavity and, under ultrasound guidance, removes residual tissue without damaging the uterus. Postoperatively, instruments are removed, an ultrasound recheck is performed to ensure complete evacuation, and appropriate postoperative care is provided.

### 2.6 Local MTX injection followed by US-guided D&C (MTX+USg-D&C)

The perineal area is disinfected, and under ultrasound guidance, a puncture needle is inserted transvaginally into the gestational sac to administer methotrexate (MTX). After observing no abnormalities, ultrasound-guided curettage is performed to remove the affected gestational tissue, with real-time monitoring of instrument position and uterine wall status to ensure thorough evacuation and prevent uterine injury. Postoperatively, instruments are removed, a check for residual tissue is performed, and the patient undergoes postoperative observation and care.

### 2.7 Hysteroscopic resection of gestational tissue (hysteroscopic resection)

The patient is carefully placed in the lithotomy position. The vulva and vagina are disinfected, sterile drapes are applied, and the uterus is distended before inserting a hysteroscope to examine the uterine cavity. After locating the gestational tissue, micro-instruments are introduced via the hysteroscope’s channel to precisely excise the tissue and its attachments, avoiding damage to normal tissues. Post-resection, the uterine cavity is checked for no residual tissue or bleeding, the hysteroscope is withdrawn, and the patient is monitored postoperatively for vital signs and vaginal bleeding, with appropriate nursing and treatment guidance provided.

### 2.8 Laparotomy with excision of gestational tissue and uterine scar repair (LT+scar repair)

General anesthesia is administered, and the patient is placed in the supine position. The area from the xiphoid process to the pubic symphysis and both sides to the mid-axillary lines is disinfected and draped. A transverse incision is made two fingerbreadths above the pubic symphysis, and the abdomen is entered. The uterus is examined to determine the location of the gestational tissue within the uterine scar. An incision is made on the uterine scar, avoiding major vessels, and the gestational tissue is carefully removed. The scar is trimmed, necrotic tissue is cleared, and the uterine muscle layers are sutured in layers with absorbable thread to achieve hemostasis and restore uterine anatomy. After confirming no intra-abdominal bleeding and verifying instrument counts, the abdomen is closed in layers. Postoperatively, the removed tissue is sent for pathological examination, and the patient is monitored for vital signs, vaginal bleeding, and wound condition.

### 2.9 Observation and post-discharge follow-up metrics

During hospitalization, dynamically monitor serum β-hCG levels, hepatorenal function, complete blood count, and CSP lesion size. Record surgical blood loss and complications (e.g., fever, vomiting, dizziness, pain). Vaginal bleeding is graded as none, minimal, moderate, or heavy, relative to menstrual flow. Within 3 months post-discharge, follow up every month. Follow-up includes serum β-hCG, hepatorenal function, complete blood count, abdominal ultrasound, and menstrual resumption. Grade complications using the NCI-CTCAE V3.0 criteria.

### 2.10 Statistical processing

SPSS 27.0 statistical software was used for data analysis. Among the data of patients with three magnetic resonance imaging (MRI) types, those who did not undergo uterine artery embolization (UAE) before termination of pregnancy were selected for multiple linear regression analysis. Taking intraoperative blood loss as the dependent variable, and “age, number of cesarean sections, number of previous uterine curettages, interval between cesarean section and pregnancy, duration of amenorrhea, amount of vaginal bleeding before admission, duration of vaginal bleeding, β-human chorionic gonadotropin (β-HCG) level, presence or absence of fetal heart activity, whether the gestational sac morphology protrudes toward the bladder on MRI images, thickness of the thinnest part of the scar, gestational sac area, gestational sac being cystic or cystic-solid, and surgical termination of pregnancy” as independent variables. Prior to conducting the multiple linear regression analysis, the dependent variable, intraoperative blood loss, underwent Log-Transformation. This log-transformation effectively converted the highly skewed distribution into a distribution that is sufficiently close to normal, thereby satisfying the residual normality assumption of the linear regression model. We have further generated and reported the results of a simplified model developed using stepwise regression. In patient data, within each of the three types, blood loss was compared and analyzed between patients who did not undergo preoperative UAE and those who had undergone preoperative UAE. For measurement data with non-normal distribution, the two independent samples rank sum test (Mann-Whitney U test) was used. *P*-value < 0.05 was considered statistically significant.

## 3 Results

### 3.1 MRI classification and patient data of CSP patients

A total of 98 patients were included (Table 1). CSP Type I: 35 patients. MRI mainly showed simple cystic images, such as ovoid, teardrop - shaped or dumbbell - shaped. The gestational sac was implanted in the uterine scar, growing toward the isthmus or cavity. Few vascular flow - voids were seen on MRI. All gestational sacs were cystic and none bulged toward the bladder. CSP Type II: 35 patients. The gestational sac could be cystic or mixed cystic - solid. Its area was larger than that of Type I, while the scar thickness was similar. The gestational sac was enhanced, with a few flow - void vessels around. However, there were no obvious vascular lakes, arteriovenous fistulas, pseudoaneurysms or aneurysms. CSP Type III: 28 patients. The time since last menstrual period (LMP) duration and vaginal bleeding duration were longer than the first two types. A higher proportion of patients had mixed cystic - solid gestational sacs, which were larger than the first two types and significantly enhanced. Flow - void vessels were visible around, along with one or more of vascular lakes, arteriovenous fistulas, pseudoaneurysms or aneurysms. Type III patients had more bleeding. All surgeries were successful, and all patients preserved their uterus. One week post - operation, β - HCG levels dropped by over 50% among all patients. Menstruation resumed in 24–47 days post - operation, with no significant difference among Type 3 patients.

**TABLE 1:** Patient characteristics.

Characteristics	Type I	Type II	Type III
Number of patients (*n*)	35	35	28
Age (year)	33 (27–43)	37 (25–44)	35 (23–44)
Number of cesarean sections (*n*)	1 (1–2)	1 (1–2)	1.5 (1–3)
Number of uterine curettages (*n*)	0 (0–1)	0 (0–2)	1 (0–3)
Interval between last cesarean section and pregnancy (year)	5 (1–17)	6 (1–17)	4 (0.8–14)
Duration of amenorrhea (day)	44 (34–63)	46 (36–74)	59 (28–84)
**Vaginal bleeding (*n*)**			
None	18	19	10
Mild	17	16	15
Moderate	0	0	2
Severe	0	0	1
Duration of vaginal bleeding (day)	0 (0–21)	0 (0–47)	15 (0–90)
β-HCG (IU/L)	17656 (1952.66–55770.9)	37561.5 (4678–172036.37)	39122.26 (3115.81–225000)
**Fetal heart activity (*n*)**			
Yes	30	34	28
No	5	1	0
**Protrusion of gestational sac toward the bladder (*n*)**			
Yes	0	2	6
No	35	33	22
Thickness of the thinnest part of the scar (mm)	1.5 (1.0–3.9)	1.5 (1.0–4.0)	2.0 (1.0–4.0)
Gestational sac area (cm^2^)	2.66 (0.96–31.28)	7.56 (2.47–60.6)	23.63 (4.32–104.5)
**Gestational sac type (*n*)**			
Cystic	31	19	1
Cystic-solid	4	16	27
**Preoperative UAE (*n*)**			
Yes	3	6	11
No	32	29	17
**Surgical termination of pregnancy (*n*)**			
USg-D&C	27	29	20
MTX+USg-D&C	7	5	3
Hysteroscopic resection	1	1	3
LT+scar repair	0	0	2
Intraoperative blood loss during termination of pregnancy (mL)	20 (10–100)	50 (5–250)	265 (10–1200)
β-HCG decrease > 50% one week postoperatively (%)	100%	100%	100%
Time to menstrual recovery postoperatively (day)	32 (25–42)	34 (24–42)	34 (24–47)

The data in the table is presented as median (minimum−maximum) or number of cases (*n*). Fetal heart activity refers to the ultrasound examination showing the embryonic heart activity in the gestational sac. UAE, uterine artery embolization; MTX, methotrexate; β-HCG, β-human chorionic gonadotrophin; USg-D&C, ultrasound-guided dilation & curettage; MTX + USg-D&C, local MTX injection followed by US-guided D&C; Hysteroscopic resection, hysteroscopic resection of gestational tissue; LT + scar repair, laparotomy with excision of gestational tissue and uterine scar repair.

### 3.2 Analysis of factors associated with blood loss during termination of pregnancy in patients with CSP

A total of 78 patients who did not undergo uterine artery embolization (UAE) before pregnancy termination surgery were included, as shown in Supplementary Table 1. We conducted a multiple linear regression analysis, with surgical blood loss as the dependent variable, and the following as independent variables to calculate risk factors associated with surgical blood loss: age, number of cesarean sections, number of previous curettages, interval between cesarean section and current pregnancy, duration of amenorrhea, amount of vaginal bleeding before admission, duration of vaginal bleeding, β-human chorionic gonadotropin (β-HCG) level, presence or absence of fetal cardiac activity, whether the gestational sac protrudes toward the bladder on magnetic resonance imaging (MRI), thickness of the thinnest part of the scar, gestational sac area, whether the gestational sac is cystic or cystic-solid, and surgical method for pregnancy termination. Supplementary Table 2 shows that the R^2^ is 0.845, indicating that the statistical results can explain 84.5% of the factors associated with surgical blood loss during pregnancy termination. The analysis results are statistically significant, as shown in Supplementary Table 3. According to the results of the multiple linear analysis, the significance was *p* < 0.001 when the gestational sac was classified as Type III (Supplementary Table 4). MRI classification of the gestational sac is an indicator for predicting surgical blood loss during pregnancy termination, and Type III on MRI is the main factor contributing to bleeding. Due to the small sample size and large number of independent variables, we generated and reported the results of a simplified model developed using stepwise regression to avoid overfitting (Table 2), and the R^2^ is 0.733. The results of the stepwise linear regression analysis demonstrated that, among all risk factors, Type III (high-risk type) of the MRI-based classification was the strongest independent predictor of intraoperative blood loss (β = 0.911, *p* < 0.001), followed by Type II (β = 0.279, *p* < 0.001), suggesting that the MRI-based classification system can reliably predict the risk of hemorrhage.

**TABLE 2:** Results of stepwise linear regression analysis of risk factors related to intraoperative blood loss.

Coefficients^a^								
Model		Unstandardized coefficients		Standardized coefficients	t	Sig.	Collinearity statistics	
		B	Std. error	Beta			Tolerance	VIF
3.000	(Constant)	1.333	0.063		21.178	0.000		
	MRI type III	1.411	0.102	0.911	13.847	0.000	0.846	1.182
	MRI type II	0.376	0.089	0.279	4.250	0.000	0.846	1.182
	MTX + USg-D&C	−0.213	0.098	−0.131	−2.166	0.034	0.999	1.002

*^a^*Dependent variable: Intraoperative blood loss during termination of pregnancy (mL).

### 3.3 Relationship between preoperative UAE and blood loss during termination surgery in three types of CSP

**FIGURE 2:**
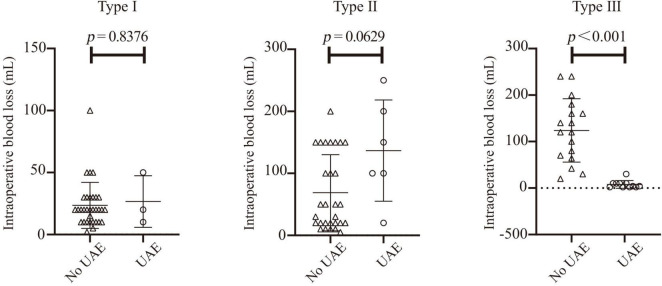
Effect of preoperative uterine artery embolization (UAE) on intraoperative blood loss in patients with three magnetic resonance classification types.

Intra-group comparisons were performed for patients with three types of CSP, and the Mann-Whitney U test (two independent samples rank sum test) was used to analyze the impact of UAE on intraoperative blood loss during pregnancy termination surgery in patients with the three types (Figure 2). Among them, there were 35 patients with MRI-classified CSP Type I, 3 of whom underwent preoperative UAE, with *p* = 0.8376. For 35 patients with CSP Type II, 6 underwent preoperative UAE, with *p* = 0.0629. Given the limited dataset of this research, preoperative UAE did not demonstrate any statistically significant advantages in either Type I or Type II patients. Due to the small number of patients who received preoperative UAE in these two types, this conclusion is unreliable and requires further statistical analysis with an expanded sample size. There were 28 patients with MRI-classified CSP Type III, including eleven who underwent UAE before pregnancy termination surgery and 17 who did not. Statistical analysis showed *p* < 0.001, indicating that preoperative UAE can significantly reduce intraoperative blood loss in patients with MRI-classified CSP Type III. For patients with Type II, due to the small sample size, no statistically significant difference was observed, and further research with an expanded sample size is required.

### 3.4 Uterine artery embolization (UAE) can bring significant benefits to patients with type III cesarean scar pregnancy (CSP) in termination of pregnancy surgery

A case in our center is as follows: a 34-year-old female was admitted due to “58 days of amenorrhea, and B-ultrasound suggesting incisional scar pregnancy for 1 day.” She had a history of two cesarean sections. Color Doppler ultrasound 2 days before admission showed “anterior isthmic pregnancy without cardiac activity (measuring approximately 36 mm × 35 mm × 30 mm, with abundant blood flow signals, 0.7 mm from the serosal layer).” She was admitted with a tentative diagnosis of “cesarean scar pregnancy.” Upon examination, her β-HCG was 154552.88 IU/L. Magnetic resonance imaging (MRI) showed that the gestational sac was cystic-solid on T2-weighted images, with a large number of vascular flow voids around the gestational sac; partial enhancement within the gestational sac was observed on T1-weighted contrast-enhanced images. Angiography under digital subtraction angiography (DSA) revealed retention of contrast agent in the pseudoaneurysm within the gestational sac. On the second day after UAE, the patient underwent local injection of methotrexate (MTX) under ultrasound guidance combined with uterine curettage, with intraoperative blood loss of approximately 20 mL. The operation went smoothly, and the patient returned to the ward safely after surgery. One week after surgery, her β-HCG was 1993.41 IU/L, and menstruation resumed on the 34th day after surgery.

## 4 Discussion

As a rare yet potentially dangerous form of ectopic pregnancy, the classification, diagnosis, and treatment of cesarean scar pregnancy (CSP) vary in emphasis across guidelines from different countries. While the American College of Obstetricians and Gynecologists (ACOG) does not have a specific classification for CSP, it addresses the condition under ectopic and high-risk pregnancies. ACOG recommends using a combination of high-resolution ultrasound and magnetic resonance imaging (MRI) to distinguish CSP from other abnormal pregnancy types and suggests early surgical intervention or medical therapy (10). The European Board and College of Obstetrics and Gyna ecology (EBCOG) has proposed a preliminary CSP classification: Type I: Gestational sac located at the uterine scar without complete myometrial penetration; Type II: Gestational sac penetrating the myometrium and invading the bladder or other surrounding structures (11). EBCOG’s diagnostic criteria emphasize imaging features and advocate for the combined use of ultrasound and MRI. Although concise and practical, this classification lacks granularity for non-penetrating myometrial cases. The Japan Society of Obstetrics and Gynecology (JSOG) introduced superficial and deep invasion subtypes based on the degree of scar invasion (12). This anatomy-focused classification is particularly valuable for guiding surgical strategies in deeply invasive cases. The Chinese Medical Association has established a three-tiered classification system: Type I: Confined to the scar; Type II: Invading the uterine myometrium; Type III: Extending to the serosa or adjacent organs (13). This more detailed classification provides a basis for individualized treatment. The Timor-Tritsch system classifies CSP categorizing cesarean scar pregnancies as on-scar cesarean scar pregnancy (oCSP) and cesarean scar ectopic pregnancy (CSeP), distinguishes the clinical presentation and risk stratification. However, its reliance on ultrasound parameters often limits its ability to comprehensively evaluate invasive vascular characteristics (14). While these consensus guidelines offer critical clinical guidance for CSP management, they still fall short of meeting practical clinical needs. There is a notable lack of effective preoperative prediction models for major hemorrhage and limited research on the optimal timing of interventional therapies like uterine artery embolization (UAE) and their role in comprehensive treatment strategies.

**FIGURE 3:**
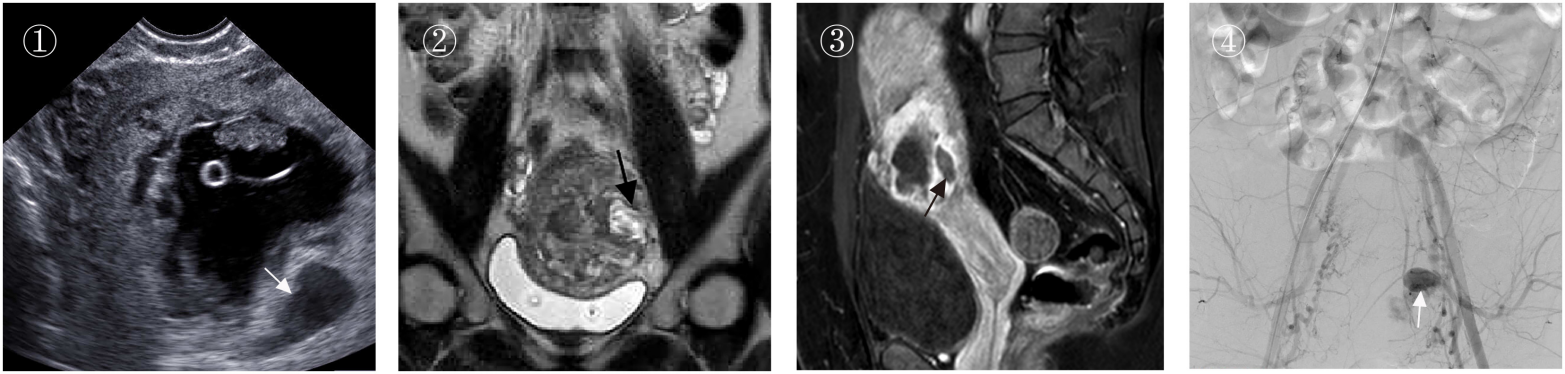
A case of type III cesarean scar pregnancy (CSP). (1). Preoperative color Doppler flow imaging ultrasound (CDFI-US) showed that the gestational sac presented a cystic-solid appearance, with an embryonic bud visible inside the sac, and a pseudoaneurysm (indicated by the white arrow); (2). Coronal T2-weighted imaging (T2WI) revealed the cystic-solid gestational sac, with multiple tortuous blood vessels around it, and a pseudoaneurysm (indicated by the black arrow); (3). Contrast-enhanced sagittal T1-weighted imaging (T1WI) showed cystic-solid enhancement within the gestational sac, with multiple tortuous blood vessels around the sac, as well as a pseudoaneurysm and its flow void effect (indicated by the black arrow); (4). Digital subtraction angiography (DSA) of the right uterine artery demonstrated the blood supply to the gestational sac, with visualization of the pseudoaneurysm (indicated by the white arrow) and the blood vessels around the sac.

Currently, ultrasound remains the primary diagnostic modality for CSP due to its non-invasiveness, cost-effectiveness, and ability to provide real-time dynamic imaging, including assessing gestational sac blood flow via color Doppler flow imaging (CDFI) (15). However, ultrasound interpretation is operator-dependent, and inexperienced sonographers may miss or misdiagnose CSP. In early or complex invasive cases, ultrasound may struggle to clearly delineate the relationship between the gestational sac, uterus, and adjacent organs (16). MRI serves as a valuable adjunct to ultrasound, particularly in complex cases (15). The most important incremental diagnostic benefit of magnetic resonance imaging (MRI) is the precise visualization of the high-risk vasculature. Our results confirm that these vascular features, uniquely captured by MRI, are the strongest independent predictors of intraoperative hemorrhage. It accurately depicts scar architecture, the extent of gestational sac invasion, and adjacent organ involvement. With superior soft-tissue contrast and multiplanar imaging capabilities, MRI is indispensable for preoperative planning in complex CSP cases (17). As MRI technology becomes more accessible in primary care settings, its use in preoperative CSP evaluation is growing, providing critical information for surgical decision-making. Emerging technologies such as 3D ultrasound and contrast-enhanced ultrasound are also being explored for preoperative CSP diagnosis. Future integration of multimodal imaging, combining ultrasound and MRI, may enable more comprehensive CSP assessments. While our classification system, particularly Type III, typically indicates hypervascularity closely associated with an increased risk of massive intraoperative hemorrhage, we present a representative case (Figure 3) that clearly illustrates the features of our MRI-based classification alongside the presentation of a pseudoaneurysm confirmed by DSA, further emphasizing the clinical significance of this high-risk characteristic and the necessity of preoperative vascular assessment. Additionally, artificial intelligence (AI)-assisted diagnosis shows promise for improving the efficiency and accuracy of CSP diagnosis and guiding treatment through large-scale image analysis and machine learning (18).

Uterine artery embolization (UAE) reduces intraoperative and postoperative hemorrhage by occluding uterine blood flow, thereby facilitating subsequent surgical interventions. While UAE is commonly used for emergency hemostasis in uncontrollable bleeding, it also serves as a preoperative adjunct for CSP, particularly in high-risk patients, to minimize the risk of massive hemorrhage. In a single-center study, UAE combined with D&C achieved a 100% treatment success rate, which underscores the importance of UAE (19). However, UAE carries inherent risks, including potential ovarian artery occlusion and subsequent ovarian function impairment in cases where ovarian and uterine arteries share a common trunk (20). Contraindications include active infection and contrast allergies. With advancements in intelligent catheter systems and robotic-assisted interventions, UAE is poised to achieve greater precision, efficiency, and safety (21).

Cesarean Scar Pregnancy is a relatively rare form of ectopic pregnancy, and this study is a single-center retrospective analysis. Although the seven-year recruitment period (from May 2016 to September 2023) indicates substantial clinical efforts, the final cohort size of 98 patients remains relatively small compared with multicenter studies. In our study, the non-significant results observed in Type I and Type II patients are most likely Type II errors (false-negative results), and do not constitute evidence to prove that UAE is ineffective in these low-risk populations. Further confirmation of this conclusion requires additional clinical data. We explicitly acknowledge that the initial full model contained an excessive number of variables, which theoretically carried a risk of overfitting. Therefore, we adopted stepwise regression linear statistical analysis. Future large-scale studies are required to validate the predictive accuracy of the simplified model. Most robust and clinically actionable finding of this study is that the benefits of UAE in Type III (high-risk) patients are extremely significant and statistically conclusive, with the median blood loss drastically reduced from 600 to 20 mL.

## 5 Conclusion

In conclusion, our analysis of MRI and clinical data from 98 patients enabled the development of a three-tiered MRI-based classification system emphasizing vascular characteristics and hemorrhage risk. This system provides practical guidance for clinical decision-making, with type III CSP identified as the strongest independent predictor of intraoperative blood loss. Subgroup analysis demonstrated that prophylactic UAE confers significant hemostatic benefit in type III cases, whereas evidence for type II remains inconclusive and requires further validation. Although the retrospective, single-center design and limited sample size restrict the generalizability of our findings, these results highlight the value of MRI classification in risk stratification and individualized treatment planning. Larger multicenter prospective studies are warranted to confirm and refine this framework.

## Data Availability

The original contributions presented in this study are included in this article/Supplementary material, further inquiries can be directed to the corresponding authors.

## Supplementary material

Supplementary Figure 1 Operational procedure of uterine artery embolization.
