# Supplementary material for: Magnetic resonance imaging–based classification of cesarean scar pregnancy: prediction of intraoperative blood loss and the role of preoperative uterine artery embolization
Source: Front Med (Lausanne). 2026 Feb 9;13:1734573. doi: 10.3389/fmed.2026.1734573 (PMC12926364; doi:10.3389/fmed.2026.1734573)
Supplement: Supplementary file 1 [file Table_1.docx]

Supplemental Table 1. Characteristics of 78 patients who did not undergo UAE.

|  | **Type Ⅰ** | **Type Ⅱ** | **Type Ⅲ** |
| --- | --- | --- | --- |
| **Number of patients (n)** | 32 | 29 | 17 |
| **Age (year)** | 33 (27-43) | 37 （25-44） | 36 (23-44) |
| **Number of cesarean sections (n)** | 1.5 （1-2） | 1 （1-2） | 1 (1-3) |
| **Number of uterine curettages (n)** | 0 （0-1） | 0 （0-2） | 1 (0-3) |
| **Interval between last cesarean section and pregnancy (year)** | 5 （1-17） | 6 （1-17） | 4 (1-14) |
| **Duration of amenorrhea (day)** | 44（34-63） | 46 （36-72） | 54 (28-84) |
| **Vaginal bleeding (n)** |  |  |  |
| None | 15 | 15 | 5 |
| Mild | 17 | 14 | 10 |
| Moderate | 0 | 0 | 1 |
| Severe | 0 | 0 | 1 |
| **Duration of vaginal bleeding (day)** | 0 (0-15) | 0 (0-22) | 20(0-50) |
| **β-HCG (IU/L)** | 17388.72 （1952.66-55770.9） | 30074 （4678-172036.37） | 19370.21 (3115.81-225000) |
| **Fetal heart activity (n)** |  |  |  |
| Yes | 27 | 28 | 17 |
| No | 5 | 1 | 0 |
| **Protrusion of gestational sac toward the bladder (n)** |  |  |  |
| Yes | 0 | 0 | 4 |
| No | 32 | 29 | 13 |
| **Thickness of the thinnest part of the scar (mm)** | 2.0 (1.0-5.0) | 2.0 (1.0-4.0) | 2.0 (1.0-4.0) |
| **Gestational sac area (cm^2^)** | 7.56 （2.47-60.6） | 6.65 （2.47-15.54） | 24.7 （5.25-104.5） |
| **Gestational sac type (n)** |  |  |  |
| Cystic | 31 | 19 | 1 |
| Cystic-solid | 1 | 10 | 16 |
| **Surgical termination of pregnancy (n)** |  |  |  |
| USg-D&C | 24 | 23 | 11 |
| MTX + USg-D&C | 7 | 5 | 3 |
| Hysteroscopic resection | 1 | 1 | 2 |
| LT + scar repair | 0 | 0 | 1 |
| **Intraoperative blood loss during termination of pregnancy (mL)** | 20 （10-100） | 50（5-200） | 600（100-1200） |
| **β-HCG decrease > 50% one week postoperatively (%)** | 100% | 100% | 100% |
| **Time to menstrual recovery postoperatively (day)** | 32 (25-42） | 34 （24-42） | 34（25-47） |

The data in the table is presented as median (minimum - maximum) or number of cases (n). Fetal heart activity refers to the ultrasound examination showing the embryonic heart activity in the gestational sac. UAE, Uterine Artery Embolization; MTX, Methotrexate; β-HCG, β-human chorionic gonadotrophin; USg-D&C, Ultrasound-guided dilation & curettage; MTX + USg-D&C, Local MTX injection followed by US-guided D&C; Hysteroscopic resection, Hysteroscopic resection of gestational tissue; LT + scar repair, Laparotomy with excision of gestational tissue and uterine scar repair.
