# Supplementary material for: Magnetic resonance imaging–based classification of cesarean scar pregnancy: prediction of intraoperative blood loss and the role of preoperative uterine artery embolization
Source: Front Med (Lausanne). 2026 Feb 9;13:1734573. doi: 10.3389/fmed.2026.1734573 (PMC12926364; doi:10.3389/fmed.2026.1734573)
Supplement: Supplementary file 2 [file Table_2.docx]

Supplemental Table 2. Results of multiple linear regression analysis on risk factors related to intraoperative blood loss volume

| **Model Summary^b^** | | | | | |
| --- | --- | --- | --- | --- | --- |
| Model | R | R Square | Adjusted R Square | Std. Error of the Estimate | Durbin-Watson |
| 1 | .920^a^ | 0.845 | 0.787 | 133.685 | 1.756 |
| a. Predictors: (Constant), Age, Number of cesarean, Number of uterine curettages, Interval between last cesarean section and pregnancy, Duration of amenorrhea, Vaginal bleeding (Mild), Vaginal bleeding (Moderate), Vaginal bleeding (Severe), Duration of vaginal bleeding, β-HCG, Fetal heart activity (Yes), Protrusion of gestational sac toward the bladder (Yes), Thickness of the thinnest part of the scar, Gestational sac area, Gestational sac type (Cystic-solid), MTX + USg-D&C, Hysteroscopic resection, LT + scar repair , MRI typeⅡ, MRI type Ⅲ. | | | | | |
| b. Dependent Variable: Intraoperative blood loss during termination of pregnancy | | | | | |
