# Supplementary material for: Magnetic resonance imaging–based classification of cesarean scar pregnancy: prediction of intraoperative blood loss and the role of preoperative uterine artery embolization
Source: Front Med (Lausanne). 2026 Feb 9;13:1734573. doi: 10.3389/fmed.2026.1734573 (PMC12926364; doi:10.3389/fmed.2026.1734573)
Supplement: Supplementary file 3 [file Table_3.docx]

Supplemental Table 3. Results of multiple linear regression analysis on risk factors related to intraoperative blood loss volume

| **ANOVA^a^** | | | | | | |
| --- | --- | --- | --- | --- | --- | --- |
| Model | | Sum of Squares | df | Mean Square | F | Sig. |
| 1 | Regression | 5379094.434 | 21 | 256147.354 | 14.333 | *p*＜.0001 |
|  | Residual | 982936.735 | 55 | 17871.577 |  |  |
|  | Total | 6362031.169 | 76 |  |  |  |
| a. Dependent Variable: Intraoperative blood loss during termination of pregnancy | | | | | | |
| b. Predictors: (Constant), Age, Number of cesarean sections, Number of uterine curettages, Interval between last cesarean section and pregnancy, Duration of amenorrhea, Vaginal bleeding (Mild), Vaginal bleeding (Moderate), Vaginal bleeding (Severe), Duration of vaginal bleeding, β-HCG, Fetal heart activity (Yes), Protrusion of gestational sac toward the bladder (Yes), Thickness of the thinnest part of the scar, Gestational sac area, Gestational sac type (Cystic-solid), MTX + USg-D&C, Hysteroscopic resection, LT + scar repair , MRI typeⅡ, MRI type Ⅲ. | | | | | | |
