# Supplementary material for: Magnetic resonance imaging–based classification of cesarean scar pregnancy: prediction of intraoperative blood loss and the role of preoperative uterine artery embolization
Source: Front Med (Lausanne). 2026 Feb 9;13:1734573. doi: 10.3389/fmed.2026.1734573 (PMC12926364; doi:10.3389/fmed.2026.1734573)
Supplement: Supplementary file 4 [file Table_4.docx]

Supplemental Table 4. Results of multiple linear regression analysis on risk factors related to intraoperative blood loss volume.

| **Coefficients^a^** | |  |  |  |  |  |  |  |
| --- | --- | --- | --- | --- | --- | --- | --- | --- |
| **Model** |  | **Unstandardized Coefficients** | | **Standardized Coefficients** | **t** | **Sig.** | **Collinearity Statistics** | |
|  |  | **B** | **Std. Error** | **Beta** |  |  | **Tolerance** | **VIF** |
| **1** | **(Constant)** | -21.578 | 208.767 |  | -0.103 | 0.918 |  |  |
|  | **Age (year)** | 2.074 | 4.5 | 0.033 | 0.461 | 0.647 | 0.542 | 1.845 |
|  | **Number of cesarean sections (n)** | -46.482 | 38.371 | -0.089 | -1.211 | 0.231 | 0.523 | 1.913 |
|  | **Duration of amenorrhea (day)** | -1.184 | 2.09 | -0.047 | -0.566 | 0.573 | 0.417 | 2.4 |
|  | **Duration of vaginal bleeding (day)** | 2.918 | 3.767 | 0.107 | 0.775 | 0.442 | 0.148 | 6.775 |
|  | **Interval between last cesarean section and pregnancy (year)** | -2.414 | 5.316 | -0.031 | -0.454 | 0.651 | 0.586 | 1.706 |
|  | **β-HCG (IU/L)** | 0 | 0.001 | -0.018 | -0.248 | 0.805 | 0.516 | 1.938 |
|  | **Number of uterine curettages (n)** | 36.04 | 54.133 | 0.081 | 0.666 | 0.508 | 0.188 | 5.332 |
|  | **Thickness of the thinnest part of the scar (mm)** | 4.671 | 19.576 | 0.015 | 0.239 | 0.812 | 0.683 | 1.464 |
|  | **Gestational sac area (cm^2^)** | -2.187 | 1.951 | -0.131 | -1.121 | 0.267 | 0.205 | 4.888 |
|  | **Vaginal bleeding (Mild)** | -68.469 | 51.845 | -0.119 | -1.321 | 0.192 | 0.345 | 2.895 |
|  | **Vaginal bleeding (Moderate)** | 269.745 | 192.937 | 0.106 | 1.398 | 0.168 | 0.486 | 2.056 |
|  | **Vaginal bleeding (Severe)** | 366.383 | 203.476 | 0.144 | 1.801 | 0.077 | 0.437 | 2.287 |
|  | **Fetal heart activity (Yes)** | -5.365 | 66.312 | -0.005 | -0.081 | 0.936 | 0.735 | 1.361 |
|  | **MTX + USg-D&C** | -36.83 | 45.966 | -0.051 | -0.801 | 0.426 | 0.7 | 1.428 |
|  | **Hysteroscopic resection** | 89.542 | 79.54 | 0.069 | 1.126 | 0.265 | 0.745 | 1.342 |
|  | **LT + scar repair** | 271.728 | 200.681 | 0.107 | 1.354 | 0.181 | 0.45 | 2.224 |
|  | **Protrusion of gestational sac toward the bladder (Yes)** | 72.146 | 94.643 | 0.056 | 0.762 | 0.449 | 0.526 | 1.901 |
|  | **Gestational sac type (Cystic-solid)** | -41.053 | 55.678 | -0.068 | -0.737 | 0.464 | 0.329 | 3.041 |
|  | **MRI typeⅡ** | 34.504 | 43.79 | 0.057 | 0.788 | 0.434 | 0.532 | 1.881 |
|  | **MRI type Ⅲ** | 327.212 | 85.044 | 0.472 | 3.848 | ＜0.001 | 0.187 | 5.361 |
| 1. Dependent Variable: Intraoperative blood loss during termination of pregnancy（mL）   UAE, Uterine Artery Embolization; MTX, Methotrexate; β-HCG, β-human chorionic gonadotrophin; USg-D&C, Ultrasound-guided dilation & curettage; MTX + USg-D&C, Local MTX injection followed by US-guided D&C; Hysteroscopic resection, Hysteroscopic resection of gestational tissue; LT + scar repair, Laparotomy with excision of gestational tissue and uterine scar repair. | | | | | | | | |
