# Supplementary figures and images for: Magnetic resonance imaging–based classification of cesarean scar pregnancy: prediction of intraoperative blood loss and the role of preoperative uterine artery embolization
Source: Front Med (Lausanne). 2026 Feb 9;13:1734573. doi: 10.3389/fmed.2026.1734573 (PMC12926364; doi:10.3389/fmed.2026.1734573)

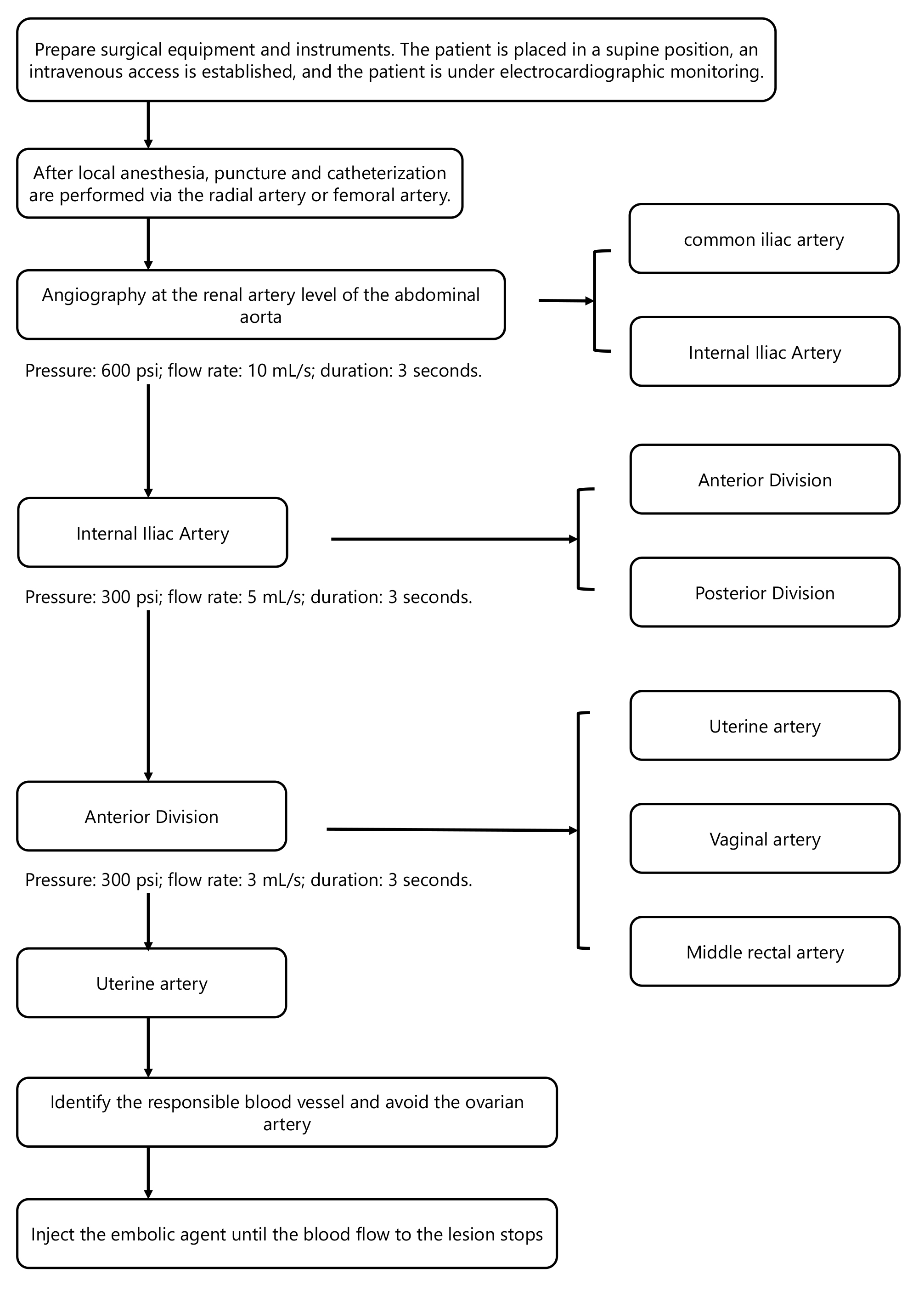

Supplement: Supplementary Figure 1 — Operational procedure of uterine artery embolization. [file Image_1.jpg]
